# Upconversion nanoparticles as intracellular pH messengers

**DOI:** 10.1007/s00216-020-02768-5

**Published:** 2020-07-02

**Authors:** Evaline S. Tsai, Fadwa Joud, Lisa M. Wiesholler, Thomas Hirsch, Elizabeth A. H. Hall

**Affiliations:** 1 Department of Chemical Engineering and Biotechnology, University of Cambridge, Philippa Fawcett Dr., Cambridge, CB3 0AS UK; 2 Cancer Research UK Cambridge Institute, University of Cambridge, LiKa Shing Centre, Robinson Way, Cambridge, CB2 0RE UK; 3 Institute of Analytical Chemistry, University of Regensburg, Universitätsstr. 31, 93053 Regensburg, Germany

**Keywords:** Upconversion nanoparticles, Nanosensors, pH measurements, Intracellular, Fluorescence microscopy, Endocytosis

## Abstract

Upconversion nanoparticles (UCNPs) should be particularly well suited for measurement inside cells because they can be imaged down to submicrometer dimensions in near real time using fluorescence microscopy, and they overcome problems, such as photobleaching, autofluorescence, and deep tissue penetration, that are commonly encountered in cellular imaging applications. In this study, the performance of an UCNP modified with a pH-sensitive dye (pHAb) is studied. The dye (emission wavelength 580 nm) was attached in a polyethylene imine (PEI) coating on the UCNP and excited via the 540-nm UCNP emission under 980-nm excitation. The UC resonance energy transfer efficiencies at different pHs ranged from 25 to 30% and a Förster distance of 2.56 nm was predicted from these results. Human neuroblastoma SH-SY5Y cells, equilibrated with nigericin H^+^/K^+^ ionophore to equalize the intra- and extracellular pH‚ showed uptake of the UCNP-pHAb conjugate particles and, taking the ratio of the intensity collected from the pHAb emission channel (565–630 nm) to that from the UCNP red emission channel (640–680 nm), produced a sigmoidal pH response curve with an apparent pK_a_ for the UCNP-pHAb of ~ 5.1. The UCNP-pHAb were shown to colocalize with LysoBrite dye, a lysosome marker. Drug inhibitors such as chlorpromazine (CPZ) and nystatin (NYS) that interfere with clathrin-mediated endocytosis and caveolae-mediated endocytosis, respectively, were investigated to elucidate the mechanism of nanoparticle uptake into the cell. This preliminary study suggests that pH indicator–modified UCNPs such as UCNP-pHAb can report pH in SH-SY5Y cells and that the incorporation of the nanoparticles into the cell occurs via clathrin-mediated endocytosis.

**Electronic supplementary material:**

The online version of this article (10.1007/s00216-020-02768-5) contains supplementary material, which is available to authorized users.

**Figure:**
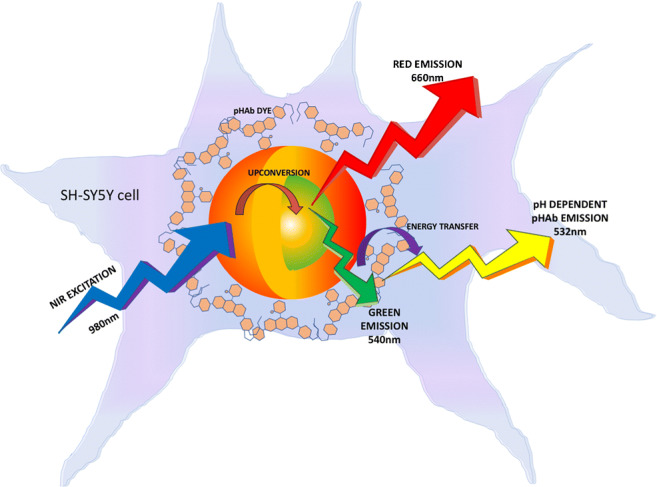
Graphical abstract

## Introduction

Dyes that have a particular affinity for the nucleus, membrane or some specific organelle in the cell can provide exquisite spatial or structural information about the cell, but it is a further challenge to obtain *spatiotemporal* information about concentrations of more mobile species like ions, metabolites and other functional molecules in the cell. By virtue of their diffusion in the cytosol, soluble dyes do not easily resolve local concentration changes, so that a route to more localized measurements has been proposed with fluorescent nanosphere or microsphere particle sensors. They could have the potential to give a real-time response to chemical and physical parameters [1–5] with high spatial resolution and negligible perturbation of the sample, but, intrinsic calibration of these particles, to turn the response into a quantitative measurement of concentration, requires a reference signal.

For this purpose, different ratiometric nanosensors using quantum dots [6–8], carbon dots [9, 10], and polymer dots [11–13] have been developed and tested that might offer an approach to intracellular metrology. For example, by exploiting fluorescence resonance energy transfer (FRET) with “passive” quantum dots embedded in the particles as reference, we have previously been successful in placing analytical nanosphere sensors (ANSor) in a human tissue culture cell line derived from embryonic kidney (HEK 293) [14] to obtain images of [H^+^] and have measured carbachol-induced intracellular calcium concentration in HeLa cells with a nanosensor incorporating calcium-selective ionophores and two fluorescence indicators to facilitate quantitative ratiometric imaging [15]. However, while these nanospheres reveal exciting new information in a model cell culture, their resolution and calibration in complex cultures is still a challenge. These studies utilized short-wavelength excitation [16] and attenuation through multiple tissue layers, autofluorescence, and photodamage is a prime hurdle to cellular concentration measurements.

Intracellular sensing of pH is of interest due to the impact of pH on cell signaling, cellular function, and other cellular mechanisms. Moving to the NIR window, which is optically transparent for biological tissue, has the potential to be a better option as the wavelength range for excitation. Wolfbeis et al. have reported a dual-sensing core-shell oxygen and pH nanosensor with the lipophilic oxygen-sensitive probe platinum (II) *meso*-tetraphenyltetrabenzoporphyrin (PtTPTBP) and the (inert) reference dye 5,10,15,20-tetrakis (pentafluorophenyl) porphyrin (TFPP) encapsulated inside a hydrophobic core and a pH-sensitive probe covalently attached to the hydrophilic shell [17]. However, excitation of these nanosensors remains in the visible region (and causes some photobleaching), but whereas the emission for the pH-sensitive probe is also in the visible, the PtTPTBP emission is in the NIR. These nanosensors do not cross the cell membrane, but can be introduced into the cytoplasm by electroporation. Nevertheless, pH values in the cytoplasm are different from in lysosomes. It is particularly important in studies of endocytosis, to be able to look at the pH when “material” is moved from outside into and through the cell. The endocytic pathway relies on vacuolar compartments that can degrade their contents upon increasing acidification. Endocytic pathways can also be used for delivery of drugs and gene therapies, as long as the cargo escapes into the cytosol before it is degraded [18, 19]. Observing the pH of these compartments is therefore critical for drug development studies.

Zhou at al have used nanospheres that have been derivatized to bind intracellular mitochondria and to accumulate in lysosomes [20]. The nanospheres contain a pH-insensitive, calcium ionophore, and they have shown that there is a burst in calcium in mitochondria after stimulation of cells using ionomycin, but [Ca^2+^] remains constant in lysosomes. Upconversion (UC)-based nanoprobes could offer further intracellular measurement. They commonly rely on sensitizer ions (e.g., Yb^3+^) and activator ions (e.g., Er^3+^, Tm^3+^) incorporated in a host matrix of NaYF_4_, NaLuF_4_, or NaGdF_4_ [21]. The emission colors and intensities of the nanocrystals can be tuned by changing the sensitizer-activator combination (e.g., Yb^3+^/Er^3+^, Yb^3+^/Tm^3+^) and dopant concentrations [22], and importantly, the emission occurs at two split wavelengths, potentially offering an inbuilt reference line for quantitative measurement of concentration.

Measurement of intracellular pH via UC nanoparticle (UCNP) has been described in the literature [18, 23–25], where the pH-sensing ability is bestowed by the presence of a pH dye, which modulates the ratio of the UCNP emission peaks depending on the pH of the surrounding environment. However, currently reported UC-based sensors fall short on mapping pH quantitatively at the subcellular level or their range does not cover the lower end of pH found in lysosomes. Two recent studies described UCNPs that were conjugated to fluorescein as pH probes, but each had their limitations. In the earlier publication, Li et al. were able to obtain a change in a ratio of 3.63 unit per unit of pH between pH 3.0–7.0 in buffers but failed to show quantitative pH-weighted images in the cells, only images of how the nanosensors colocalized with a lysosome dye in QBC939 cells [25]. In the later report by Du et al., the live cell quantitative imaging was only achievable down to pH 5.0 [24], which is above the pH attained by late endosomes and lysosomes for degrading internalized material [26, 27].

In addition, UC-based pH-sensing studies so far have not quantified colocalization with endosomes/lysosomes nor studied the endocytic method of uptake. One study by Jin et al. proposed that PEI-coated UCNPs enter mammalian cells through clathrin-mediated endocytosis [28], but it is possible that modifying the surface of the UCNP with a dye could change the uptake process. The mechanism by which the nanosensor is taken up by the cells is important because it limits the type of drug that can be tracked in drug delivery studies. For example, nanosensors that enter through the clathrin-mediated endocytosis pathway would be unable to sense pH in the endosomes/lysosomes that contain drugs entering through the caveolae-mediated endocytosis pathway.

In the work reported herein, UCNPs are investigated after conjugation to pHAb, a pH-sensitive dye that increases in fluorescence as the environment becomes more acidic. The absorption of pHAb coincides well with the green emission band of the UCNP, and the dye’s emission intensity varies between pH 4.0 to 6.0. This makes it ideal for sensing in acidic compartments like endosomes and lysosomes that are involved in endocytosis. Colocalization studies are presented to determine the subcellular location of the UCNP-pHAb nanoconjugates and pharmacological inhibitor studies are reported to help determine the mechanism by which the nanosensors are taken up by the cells.

## Materials and methods

### Materials

YCl_3_•6H_2_O and YbCl_3_•6H_2_O (both > 99.9%) were obtained from Treibacher Industrie AG. Oleic acid and 1-octadecene (both 90%) were from Alfa Aesar. NH_4_F, ErCl_3_•6H_2_O (99.99%), NaOH, polyethylenimine (PEI) (branched, average *M*_w_ ∼ 25 kDa), and nitrosyl tetrafluoroborate (NOBF_4_) (95%) were from Sigma-Aldrich.

The pHAb amine reactive dye was purchased from Promega. DMSO (99 + %) was from Alfa Aesar. Potassium chloride (99 + %) and sodium bicarbonate (99 + %) were from Acros Organics. Amine conjugation buffer was made by dissolving 0.084 g sodium bicarbonate in water, adjusting the pH to 8.5 using HCl/NaOH (100 mM), then adjusting the final volume to 100 mL with water. Citric acid monohydrate, disodium phosphate heptahydrate, and chlorpromazine hydrochloride (≥ 98%) were purchased from Sigma-Aldrich. All cell culture media were purchased from Thermo Fisher Scientific. Nigericin sodium salt was from Cayman Chemical. Cell Navigator green fluorescence, with 405 nm excitation, lysosome staining kit was purchased from AAT Bioquest. Nystatin was obtained from BioVision. The resazurin-based alamarBlue cell viability reagent and Trypan blue solution (0.4%) were purchased from Thermo Fisher Scientific. SH-SY5Y cells were obtained from the MRC Stem Cell Institute (University of Cambridge).

### Synthesis of UCNPs

The oleate-capped UCNPs were synthesized according to the high-temperature coprecipitation method as described in prior literature [29, 30]. A host matrix of NaYF_4_ and lanthanide ions of Yb^3+^ and Er^3+^ were selected for their popularity in UCNP studies to date and their emission in the green/red spectrum. A thin shell (1 nm thick) was grown to protect the UCNP from quenching effects. Core-shell particles are known to have brighter luminescence than core-only particles [31]. The synthesis protocol is described in the Electronic Supplementary Material (ESM).

### UCNP surface modification with PEI

The UCNPs were modified with PEI to enable the formation of an amide bond between UCNP and dye. A two-step ligand exchange process was used to replace the oleate on the surface of the UCNPs with PEI [31]. The nanoparticles were dispersed in a cyclohexane/DMF system with NOBF_4_ (1 mg per 1 mg UCNPs), and the resulting mixture was stirred for 20 min at 30 °C. The cyclohexane phase, which contained the free oleic acid, was removed. The BF_4_^−^ coordinated particles were precipitated by adding chloroform and separated by centrifugation (1000*g*, 5 min). The pellet was redispersed in DMF and washed once with chloroform/DMF. Lastly, the pellet was dispersed in DMF and centrifuged (1000*g*, 3 min) to eliminate aggregates. The supernatant was collected and could be stored in the dark at 4 °C if the PEI exchange process was not immediately continued.

A total of 100 mg PEI (branched, ~ 25 kDa) was dissolved in 8 mL ddH_2_O and heated to 50 °C under stirring. Forty milligrams of the UCNPs in 2 mL DMF were added dropwise to the flask. The mixture was heated to 80 °C and stirred for 1.5 h under reflux. The particles were centrifuged at 21000*g* for 1 h, washed with double distilled water, and redispersed by shaking/sonication. These wash steps were repeated thrice. Finally, the particles were filtered through a 220-nm PES filter.

### UCNP conjugation with pHAb

The pHAb amine reactive dye was quickly centrifuged (i.e., 14,000*g* using a tabletop centrifuge for 5–10 s) and dissolved at 10 mg/mL by adding 25 μ L of 1:1 DMSO-water mix. It was mixed by vortexing until the dye was dissolved completely (1–3 min). Five milligrams of the PEI-coated UCNPs in amine conjugation buffer was mixed with the pHAb amine reactive dye in DMSO-water. The mixture underwent stirring for 60 min while protected from light. During this time, the succinimidyl ester on the pHAb dye reacts with the primary amine groups of the PEI to form a stable amide bond, and the basic environment minimizes any hydrolysis side reaction of the succinimidyl ester. The UCNP-PEI-pHAb conjugate was washed three times with water. Finally, it was suspended in water and stored at 4 °C.

### Characterization of the pH Nanoprobe

An inductively coupled plasma optical emission spectrometer (ICP-OES) (Spectro) was used for the concentration determination of the nanoparticles. TEM images of the oleate-capped UCNPs were obtained with a 120 kV Philips CM12 transmission electron microscope (FEI). The images of the UCNPs were analyzed with ImageJ software (NIH) to obtain size distribution.

The zeta potential of the UCNPs was measured with a Zetasizer Nano ZS (Malvern) at room temperature. The hydrodynamic diameter and zeta potential of the UCNP-pHAb were measured with a ZetaPALS (Brookhaven Instruments) at room temperature in the BP Institute (University of Cambridge). Samples were recorded in triplicate, with separate samples taken.

Absorption measurements were performed at room temperature with a Synergy HT (BioTek). The following equation was used to calculate the dye-to-particle ratio:

$$ \frac{A_{532}\times {MW}_{particle}}{Particle\ Concentration\ \left(\frac{mg}{ml}\right)\times \mathrm{75,000}} $$

where *A*_*532*_ = absorbance of *λ*_*max*_ of pHAb, *MW*_*particle*_ = molecular weight of the UCNPs, and extinction coefficient of pHAb dye = 75,000. The spectral overlap integrals of the UCNP-pHAb pairs were calculated with a|e software (FluorTools).

Phosphate/citrate buffers of different pHs (0.2 M) were added to UCNP-pHAb solution (~ 1 mg/mL) in a 1:1 volume ratio. A Cary-Eclipse Fluorescence Spectrophotometer (Varian) was used to collect the emission spectra of UCNP-pHAb when the dye was directly excited. Instrument excitation and emission slits were both set at 5 nm, and the scan rate was 120 nm/min. All samples were excited at 532 nm, and the emission was scanned from 540 to 650 nm. The photomultiplier tube detector voltage was set at 800 V.

Other characterization spectra were enabled through the access to the NanoPhotonics Centre (University of Cambridge, see acknowledgements): a QE65000 spectrometer (Ocean Optics) was used to obtain the luminescence spectra for pH titration wherein the UCNP was directly excited at 980 nm with a Spectra-Physics Mai Tai Ti:Sapphire NIR/IR laser (Newport). A 750-nm shortpass filter (Thorlabs) protected the detector from the laser. All spectra were recorded at room temperature. Binomial filtering (50 passes) with Igor Pro software (Wavemetrics) was used to smooth the spectra because it reduces noise while maintaining peak position [32].

A setup consisting of a 980 nm cw laser module (200 mW) (Picotronic) and an optical chopper (MC2000 with two slot chopper blade MC1F2) (Thorlabs) was used to measure lifetime. The signal was intensified by a photomultiplier tube (PreSens) and analyzed with a digital storage oscilloscope (DSO 8204) (Voltcraft). Optical bandpass filters (FF01-535/150-25 and FF01-665/150-25) (Semrock) were also used. The UCNP-pHAb samples were diluted before lifetime measurement because high dye concentration caused too much of the green light to be reabsorbed to obtain a sufficiently high photon count on the PMT. The decay fits were obtained with Prism 8 (GraphPad). The average lifetime values were calculated by averaging the lifetimes obtained from three monoexponential fits of the solution of interest.

### Multiphoton imaging of the pH Nanoprobe

Two hundred microliters of agarose gel in phosphate-buffered saline (PBS) (2.5%) was mixed with 100 μL of UCNP-pHAb in water (1 mg/mL) at 90 °C. A total of 100 μL of the resulting mixture was pipetted into an 8-well ibiTreat μ-Slide (Ibidi), placed in a refrigerator until solidified, then imaged with a TCS SP5 Confocal (Leica) equipped with a pulsed Chameleon Ultra II IR laser (Coherent). Three color channels were captured using HyD hybrid detectors (Leica): 500–550 nm (UCNP green emission), 565–630 nm (pHAb yellow emission), and 640–680 nm (UCNP red emission).

### Multiphoton imaging of cells

SH-SY5Y cells were cultured at 37 °C in 5% CO_2_ in Dulbecco’s Modified Eagle Medium (DMEM) at physiological pH, supplemented with 10% fetal bovine serum (FBS) and 1% penicillin/streptomycin in a 4-well ibiTreat Ph+ μ-Slide (Ibidi). A total of 50,000 cells were plated in each well (700 μL) ~ 36 h before imaging then UCNP-PEI-pHAb conjugates in clear DMEM/F-12 (0.01 mg/mL) were added ~ 12 h before imaging. For in situ pH calibration, the intracellular pH was adjusted to that of the extracellular environment according to previously described protocols [18, 33, 34]. Briefly, the cells were treated with citric acid/phosphate buffer of certain pH supplemented with 140 mM KCl and 10 μM nigericin then returned to incubation for 10 min. Multiphoton imaging was performed on a TCS SP5 Confocal (Leica) equipped with a pulsed Chameleon Ultra II IR laser (Coherent). A CO_2_ and temperature control incubator was placed on the microscope stage for live cell imaging. Three color channels were captured using HyD hybrid detectors (Leica): 500–550 nm (UCNP green emission), 565–630 nm (pHAb yellow emission), and 640–680 nm (UCNP red emission). Cell outlines were visualized with transmission detection. Control cells were imaged after ~ 12 h of incubation with UCNP-PEI-pHAb conjugates but were not exposed to pH buffer supplemented with KCl and nigericin.

MATLAB (Mathworks) was used for image processing and analysis, which were based on previously reported methods [33–36]. After median filtering, a mask was created using a threshold obtained from Otsu’s method. Using this mask, the pHAb (yellow channel) to UCNP (red channel) intensity ratio was calculated at each pixel. The average ratios were plotted as a function of pH to obtain a calibration curve. The ratiometric images were generated after the described masking had been applied. The intensity value of each pixel in the pHAb channel (yellow) was divided by that of the corresponding pixel in the UCNP channel (red). The image pseudocolors were rescaled according to the calibration curve, and a colorbar was applied to the image.

### Confocal laser scanning imaging of cells

SH-SY5Y cells were cultured at 37 °C in 5% CO_2_ in Dulbecco’s Modified Eagle Medium (DMEM) (Thermo Fisher Scientific) supplemented with 10% fetal bovine serum (FBS) and 1% penicillin/streptomycin in a 4-well glass-bottom μ-Slide (Ibidi) coated with poly-d-lysine. Fifty thousand cells were plated in each well (700 μL) ~ 60 h before imaging then UCNP-PEI-pHAb conjugates in clear DMEM/F-12 (0.01 mg/mL) were added ~ 12 h before imaging. The lysosomal-staining solution was prepared by diluting the LysoBrite dye in the live cell staining buffer provided in the Cell Navigator kit (20 μL:10 mL). A total of 350 μL of the medium was taken out and replaced by 350 μL of dye working solution then placed in the incubator for 30 min. Confocal laser scanning microscopy was performed on a TCS SP8 (Leica) equipped with a white light laser source and 405-nm diode laser. The LysoBrite dye was excited at 405 nm, and the pHAb dye was excited at 532 nm. Photomultiplier tubes (Leica) collected emission from 480 to 520 nm (cyan channel) and 565–630 nm (yellow channel). The imaging system was enclosed to maintain the temperature at 37 °C and CO_2_ at 5% for live cell imaging.

Fiji, an open-source image processing package, and Coloc 2, a plugin, were used for colocalization analysis [37]. Using the image preprocessing and analysis methods outlined by Dunn et al. [38], background images were created for both channels in which the value of each pixel from the original image was replaced with the median intensity of a surrounding 26 × 26 (yellow channel) or 32 × 32 (cyan channel) pixel region. These background images were then subtracted pixel-by-pixel from the original image to generate a median-subtracted image. To analyze intracellular vesicles, the region of interest (ROI) was defined as the cytoplasm, excluding the extracellular space and nucleus. The two probes, LysoBrite and pHAb, were designated *C* and *Y*, respectively. Manders’ colocalization coefficients (MCCs) were then calculated for the ROI with the following equations:

$$ {MCC}_1=\frac{\sum_i{C}_{i, colocal}}{\sum_i{C}_i} $$

$$ {MCC}_2=\frac{\sum_i{Y}_{i, colocal}}{\sum_i{Y}_i} $$

where *MCC*_*1*_ is the fraction of *C* in compartments containing *Y* and *MCC*_*2*_ is the fraction of *Y* in compartments containing *C*. *C*_*i*_ and *Y*_*i*_ are the intensities of *C* and *Y*, respectively, at pixel *i*. *C*_*i,colocal*_ = *C*_*i*_ if *Y*_*i*_ > 0 and *C*_*i,colocal*_ = 0 if *Y*_*i*_ = 0. *Y*_*i,colocal*_ = 0 if *C*_*i*_ = 0.

Statistical tests were performed with Prism 8 (GraphPad) based on the significance tests of MCC measurements of colocalization described by McDonald et al. [39]. The expected MCC was the proportion of pixels above the background. This value was subtracted from the observed MCC to obtain *MCC*_*diff*_. The mean *MCC*_*diff*_ was compared with 0 using a Student’s one-tailed, one-sample *t* test, with the significance level set to 0.05.

### Endocytosis experiments

The protocol for the endocytosis experiments was adapted from a method developed by Teplensky et al. [40]. The concentrations of chlorpromazine and nystatin were based on the work by Qu et al. with SH-SY5Y cells [41].

SH-SY5Y cells were cultured before being seeded on a 24-well plate at a density of 100,000 cells/well. After approximately 24 h, the cells were washed with PBS before a pretreatment 30 min incubation with different inhibitors in DMEM/F-12 without phenol red at 37 °C: untreated (200 μL), chlorpromazine (200 μL, 10 μg/mL), nystatin (200 μL, 25 μg/mL). Afterwards, the medium was aspirated and replaced with a solution of UCNP-pHAb (0.1 mg/mL, DMEM/F-12 without phenol red) in each set of conditions: untreated (200 μL), chlorpromazine (200 μL, 10 μg/mL), nystatin (200 μL, 25 μg/mL). The plate was incubated for 1.5 h at 37 °C. After incubation, the well contents were removed and washed three times with PBS. The cells were then incubated with trypsin for 5 min at 37 °C. Medium was added to the wells to stop the trypsinization, and the contents were transferred to tubes and centrifuged at 1300 rpm for 5 min. The cells were resuspended in 300 μL of flow buffer (PBS with BSA). Samples were kept on ice until measurement with a CyAn ADP flow cytometer (Beckman Coulter). The data were analyzed with FlowJo and Prism 8 (GraphPad) software. A longer incubation time of 3 h induced a cytotoxic response for the cells treated with chlorpromazine.

### Cell viability assay with Trypan blue exclusion assay

SH-SY5Y cells were plated in two wells of a 6-well plate. After the cells were incubated at 37 °C and in 5% for 24 h, one well was treated with 2 mL of UCNP-pHAb solution (0.01 mg/mL in DMEM/F-12, no phenol red) and the other well was treated with 2 mL of DMEM/F-12 medium without phenol red. The cells were then incubated for another 24 h. The adherent cells were suspended using 2 mL of trypsin and gently triturated with a pipette to break up clumps. 0.1 mL of cell suspension was mixed with 0.1 mL of trypan blue solution. A hemocytometer chamber was filled with 15 μL of the mixture and viewed under a light microscope. The number of viable (bright cells) and non-viable cells (stained blue) was counted, and the cell viability was calculated using the following formula:

$$ Percentage\ viability=\frac{Number\ of\ viable\ cells}{Total\ number\ of\ cells}\times 100 $$

## Results and discussion

### Preparation and characterization of UCNP-pHAb sensor

As shown by us and others previously, the multiple emissions of the UCNPs at different wavelengths allow ratiometric sensing to be achieved. Depending on the chemistry of the indicator and distance from the UCNP linked via surface modification, it is possible to use one band of the UCNP (eg green emission) to excite a pH-sensitive moiety through the inner filter effect or energy transfer, while the other UCNP band (eg red emission) acts as a reference for quantitative ratiometric measurements [18, 23, 24, 31]. This could also “turn-on” a separate analyte-dependent peak relative to a reference UCNP emission band. The latter could be much more sensitive for pH sensing in cells due to lower background fluorescence. It also gives the opportunity for either the green or red band to be used in the ratio (or both) and, for the study here, for the pH-sensing dye to be excited directly or via the UCNP.

**Fig. 1:**
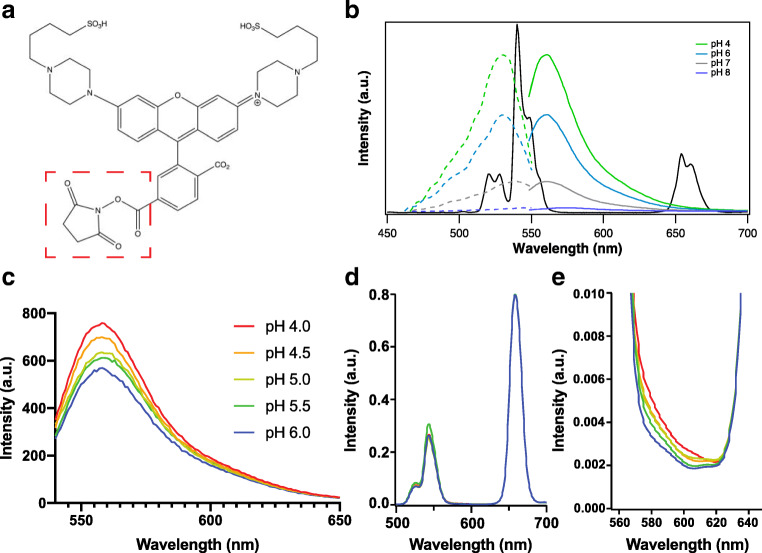
(a) Molecular structure of pHAb amine reactive dye. The succinimidyl ester group here (enclosed by red dashed box) can react with primary amines to form an amide bond. Structure created with ChemDraw (b) The absorption (dashed) and emission (solid) spectra of pHAb (in color) are overlaid with the emission spectrum of thin-shell, PEI-coated UCNP [31] upon 980 nm excitation (in black). (c) Emission spectra at various pHs when UCNP-pHAb in phosphate/citrate buffer is excited at 532 nm (direct excitation of the dye). (d) Emission spectra of UCNP-pHAb in 0.2 M phosphate/citrate buffer at pH 4.0, 4.5, 5.0, 5.5, and 6.0 upon 980 nm excitation after binomial smoothing. (e) Zoom-in of the sensitized pHAb emission spectra from (d). (d) and (e) Use the same color scheme as (c) It is noted here that Fig. 1 b and d have different intensity ratios due to the different instruments used for measurement

pHAb (Fig. 1a) was selected as the pH indicator because its absorption band overlaps well with the luminescence of Er^3+^ at 540 nm across intracellular pHs (Fig. 1b). Moreover, the pH-sensitive emission band of pHAb largely avoids the Er^3+^ luminescence at 650 nm, which functions as the reference signal. Thus, the strategy is to use the pHAb emission and red UCNP emission together in a sensing system for ratiometric intracellular pH imaging. Despite there being some overlap between the green UCNP and the 540-nm dye emission in this particular configuration, the ability to reference to the red band provides a good analytical option. By examining Fig. 1b, it can be seen that a single wavelength intensity measurement at around 575 nm or an integration from circa 565–630 nm could be selected to largely avoid cross-talk with the 540 emission and that the 540 UCNP emission is very strong, so that energy transfer to pHAb will not be limited.

**Fig. 2:**
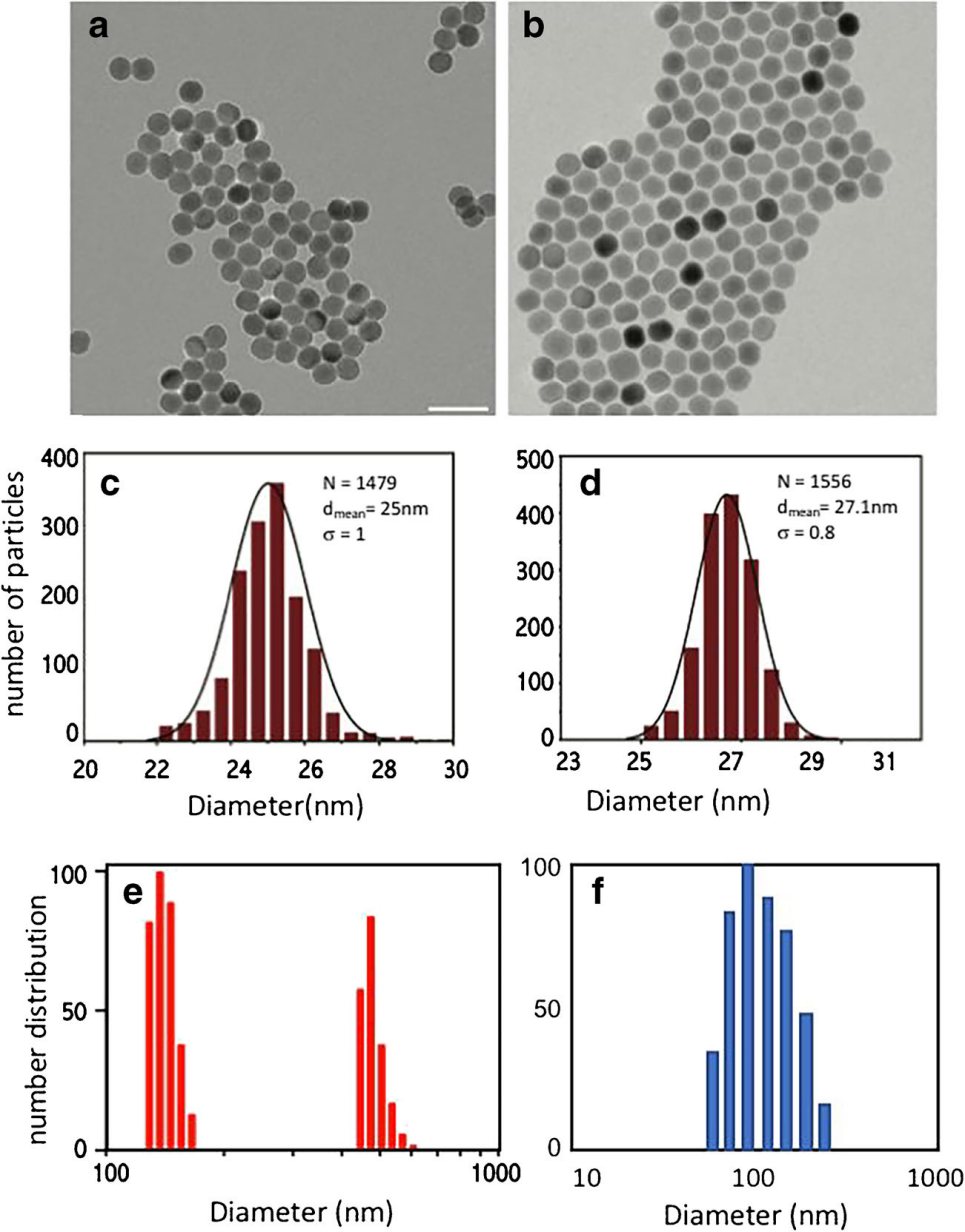
TEM images of monodisperse, oleate-capped **a** core NaYF4: 20% Yb, 2% Er and **b** core-shell NaYF4: 20% Yb, 2% Er@NaYF4. The scale bars are 60 nm. Histograms of the size distribution from the TEM images are shown in **c** and **d** for the core and core-shell particles, respectively. Number-weighted histogram of particle diameters obtained from DLS measurements of UCNP-pHAb **e** in unbuffered aqueous media were **f** buffered at pH 4.0

To produce the UCNPs, a shell layer of inactive NaYF_4_ was grown around the NaYF_4_: Yb^3+^, Er^3+^ core, to minimize the quenching effects of surface defects and solvent. The core particles had a mean diameter of 25 nm, and the thickness of the shell was ~ 1 nm (calculated from the TEM images in Fig. 2). This thickness was chosen as a compromise between the thinness required for resonance energy transfer (RET) and the thickness that reduces environmental quenching and promotes bright luminescence. Branched, high molecular weight PEI was selected as a coating for its ability to facilitate cellular uptake [18, 23] and also to bind the dye through amide coupling between the amine groups and the succinimidyl ester group of the pHAb. Covalent attachment of pHAb to the surface of UCNPs minimizes dye leaching, preventing the dye and UCNPs from localizing in different parts of the cells [24]. Successful conjugation of pHAb to the UCNP was confirmed visually, by the red color on the surface. From absorbance and ICP-OES measurements, this reaction was estimated to yield ~ 400 dye molecules per individual UCNP particle. Similar yields have been obtained by Muhr et al. [42] using an organic solvent–based exchange of capping ligand and fluorescent dye, which reduces the distance between lanthanide donor and organic dye receptor, but that method produced a more hydrophobic UCNP, which is not conducive to cellular uptake.

From DLS measurements, the UCNP-pHAb conjugates in unbuffered aqueous media were determined to have two size distribution peaks of 141 ± 10 (s.d.) nm and 481 ± 33 (s.d.) nm, of which the latter suggested significant aggregation (Fig. 2e). Aggregation, causing the bimodal distribution, was consistent with the average zeta potential of +22 mV, which was a decrease from the zeta potential of +36 mV before dye attachment [31]. Particles with zeta potentials within the range seen here may agglomerate. In contrast, UCNP-pHAb conjugates incubated in buffer at pH 4.0 produced a distribution peak for the hydrodynamic radius at 125 ± 30 (s.d.) nm and showed a zeta potential of + 32 mV. These particles were stable in suspension without agglomeration for > 12 h. Stability and zeta potential reduced with pH, but even at pH 5.5 (zeta potential + 28 mV) the conjugates showed short term colloidal stability (< 12 h). Generally, nanoparticles with a zeta potential below − 30 mV or above + 30 mV have sufficient electrostatic stabilization such that they do not aggregate quickly [18].

After conjugation to the UCNP, the pHAb dye retains its fluorescence response to pH change when directly excited at 532 nm (Fig. 1c) Moreover, there is no noticeable shift in the emission band. Furthermore Fig. 1e shows that the emission spectra of pHAb in UCNP-pHAb nanoconjugate are just discernable as a shoulder on the 540 UCNP emission (Fig. 1d), in 0.2 M citrate/phosphate buffer from pH 4.0–6.0 under 980 excitation.

The decrease in fluorescent lifetime of the 540 nm emission after conjugation with the dye (Table 1) was consistent with the UC-RET mechanism and showed that the UC-RET efficiencies (E) at different pHs ranged from 25 to 30%, according to:

$$ E=1-\frac{\tau {\left( UCNP- pHAb\right)}_{540\  nm}}{\tau {(UCNP)}_{540\  nm}} $$

**Table 1:** Lifetimes and UC-RET efficiencies of 540 nm green emission of UCNPs, diluted UCNP-pHAb, and photobleached UCNP-pHAb

	pH 4	pH 5	pH 6
UCNPs (540 nm)	356 ± 9 μs	329 ± 5 μs	359 ± 13 μs
UCNP-pHAb (540 nm)	254 ± 44 μs	248 ± 9 μs	251 ± 45 μs
UC-RET efficiency	29 ± 5%	25 ± 1%	30 ± 6%

Muhr et al. [42] found that the efficiency was a function of the surface area:volume ratio, and at similar UCNP size and dye loading to those used in this study, they could achieve up to 60% efficiency. Nevertheless, the increased donor-acceptor distance anticipated through the PEI coating is consistent with the results obtained here and a Förster distance of 2.56 nm is predicted from these results, using the following equation:

$$ {R}_0=\sqrt[6]{\frac{9\times \ln 10\times {\kappa}^2\times {Q}_D\times J}{128{\pi}^5\times {N}_A\times {n}^4}} $$

Here, *κ*^*2*^ is the dipole orientation factor, *Q*_*D*_ is the quantum yield of Er^3+^, *J* is the spectral overlap integral of the UCNP and pHAb dye, *N*_*A*_ is Avogadro’s number, and *n* is the refractive index. Similar to previous publications [31, 42–45], we assumed a *κ*^*2*^ of 2/3 (given the long lifetime of UCNPs), *Q*_*D*_ of 0.01, and *n* of 1.48 (relevant medium is NaYF_4_). We calculated a *J* of 2.3 × 10^15^ nm^4^/(M cm) for the UCNP-pHAb pair.

### Microscopy of the UCNP-pHAb Nanoprobe

Before considering in situ calibration and in vitro validation of the pH nanosensor, the UCNP-pHAb conjugate was imaged on its own in an agarose phantom. Huefner et al. [46] have suggested that intracellular AuNPs are likely to form aggregates in the cell, so a phantom to simulate this effect is desirable. Dispersion in the agarose resulted in significant aggregation of particles in the gel consistent with this finding. The aim was to confirm the feasibility of using the probe for optical imaging with a Leica SP5 confocal fluorescence microscope. “Green” (500–550 nm), “yellow” (565–630 nm), and “red” (640–680 nm) emission channels were acquired upon 980 nm excitation of UCNP-pHAb conjugate in agarose gel. These channel ranges were selected to minimize cross-talk in order to resolve the pHAb emission: at the emission maximum for pHAb at 560 nm (Fig. 1), there is still ~ 4% signal intensity of the UCNP green emission peak at that wavelength compared with ~ 0.3% at 565 nm (Fig. 1b). Each channel was calibrated between 0 and 100% intensity.

**Fig. 3:**
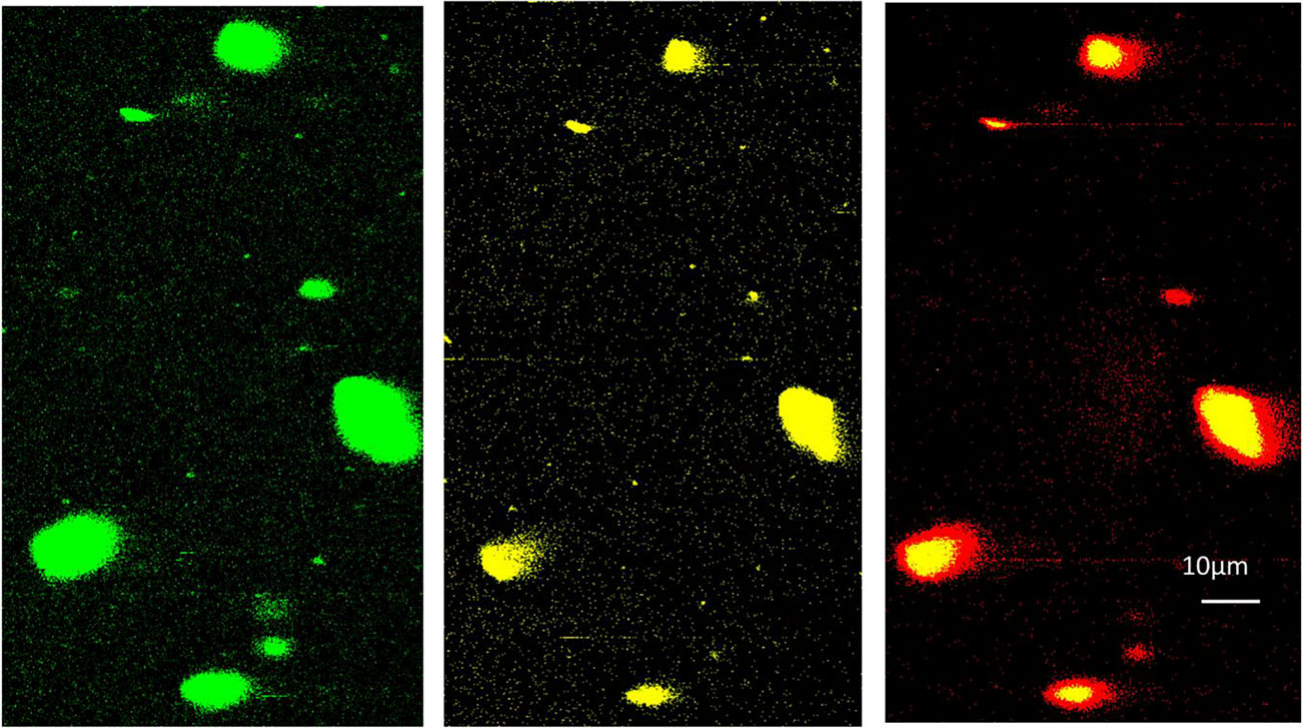
Confocal fluorescence microscope images of the green, yellow, and red emission of an area of UCNP-pHAb conjugate clusters in agarose gel. The excitation wavelength was 980 nm, and the images were collected in the ranges of 500–550 nm (UCNP green), 565–630 nm (pHAb yellow), and 640–680 nm (UCNP red). The red channel shows pixel saturation

As expected, single particles were not resolved under these conditions, but the nanosensor aggregates in agarose gel could be viewed under the microscope (Fig. 3). Since excitation was at 980 nm, the pHAb dye (yellow channel) was not directly excited at 532 nm, but via the green channel emission, and the spatial coincidence between the green and the yellow channel is consistent with resonance energy transfer from the UCNP to the dye to produce the 565–630-nm fluorescence. In the absence of dye, the emission recorded in the yellow channel was below the detection limit.

Based on these phantom results, pH might be able to be resolved within the range for the pHAb dye. However, the cytotoxicity of UCNP-pHAb nanoconjugates is also an important consideration. This was evaluated using a trypan blue exclusion assay to indicate cell viability. After incubation with 0.01 mg/mL of nanoprobes for 24 h, no significant decrease in cell viability was observed (ESM Fig. S1). In order to adjust the pH inside cells, various pH buffers containing nigericin were used. The nigericin H^+^/K^+^ ionophore equilibrates pH across the cell membranes so that the inside of the cells becomes the same pH as that of the extracellular buffer [47]. Human neuroblastoma SH-SY5Y cells were equilibrated for 12 h before measurements were recorded.

**Fig. 4:**
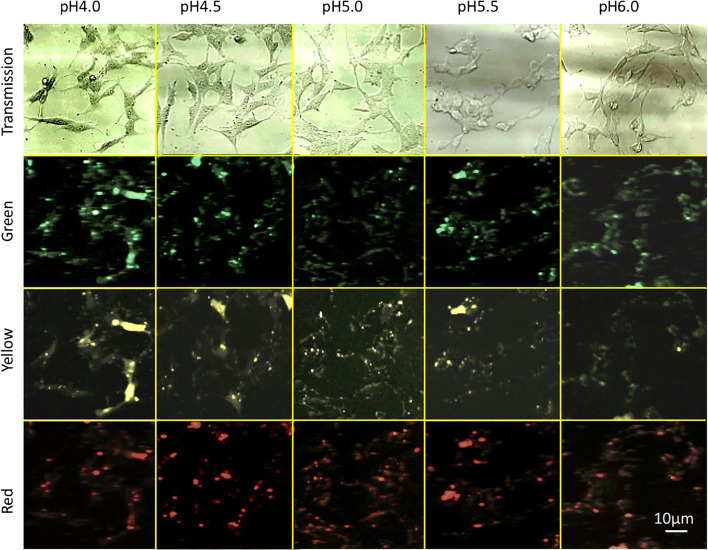
Multiphoton confocal microscopy images of UCNP-pHAb in nigericin-treated SH-SY5Y cells exposed to citric acid/phosphate buffer with KCl at pH 4.0, 4.5, 5.0, 5.5, 6.0. The excitation wavelength was 980 nm, and the images were collected with 2x frame accumulation in the ranges of 500–550 nm (UCNP green), 565–630 nm (pHAb yellow), and 640–680 nm (UCNP red). The corresponding transmission image is also shown. The images have been processed using Photoshop (Adobe). Scale bar = 10 μm

The transmission images in Fig. 4 show healthy, adherent SH-SY5Y cells characteristically extending their neurites and spreading [48–50]. The three emission channels of UCNP-pHAb conjugates with SH-SY5Y cells indicate that the UCNP-pHAb conjugates become associated with the cells. These images cannot fully resolve the spatial location of individual or clusters of particles, but the transmission images, especially at low pH suggest that some particles are located on the cell membrane without forming large clusters. At low pH, the higher surface charge from the PEI-pHAb coating on the UCNP-pHAb conjugates which mediates adhesion to the negatively charged cell surface may be correlated with the position of these particles seen in the transmission images. However, the emission images suggest a higher density of particles that overlay with the cell shape. Nanoparticles are known to be voluntarily taken up by cells dependent on factors such as charge, surface groups, size, and shape. Once in the cell, they are often trapped inside membrane-bound vesicles of the endocytotic pathway. Huefner et al. [46] report that intracellular AuNPs are likely to form aggregates inside endosomes/lysosomes.

**Fig. 5:**
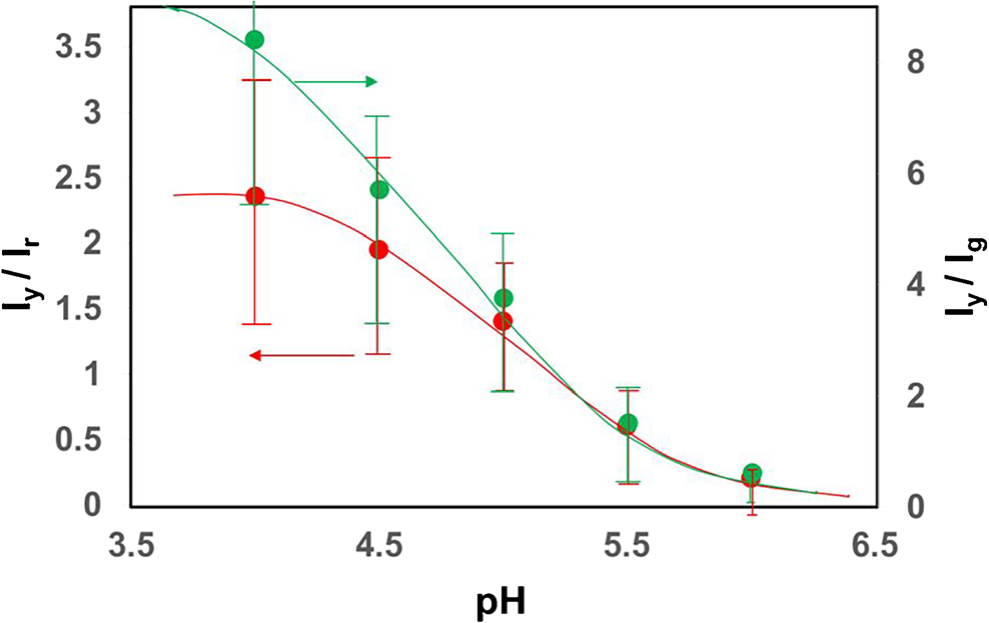
pH calibration curve of UCNP-pHAb in SH-SY5Y cells in different pH buffers supplemented with KCl and nigericin, based on the average of the ratio of (left axis) yellow (*I*y) to red (*I*r) fluorescence intensity or (right axis) yellow (*I*y) to green (*I*g) fluorescence intensity at each pixel of interest. Note: the pixel intensity contributing to the “red,” “yellow,” “green” channels do not correlate with intensity ratios obtained from Fig. 1b for selected wavelengths at the different pHs, but is made up of RBG data for that channel, calibrated between 0 (min) and 255 (max) and having arbitrary units. Data (150 samples) taken from areas locating the cells in the transmission image from duplicate samples. Sample area varied according to cell size

The fluorescence images (Fig. 4) were collected with 2-fold frame accumulation to increase overall brightness to more easily be able to visually compare the images at the various pHs. However, pH calibration was examined on images without frame accumulation to avoid saturation, which causes loss of information in the brightest areas of the sample. Each channel was scaled. When the extracellular pH changes, the fluorescence images acquired from different areas where particles were located, show a change in the ratio of the yellow/red or yellow/green channels. Sampling was focused on areas identified from the transmission image, where there were cells and bright areas of emission in the 3 channels, suggesting particle aggregation. If the aggregates correlate predominantly with intracellular particles, this suggests a consequent change of pH inside the cells: taking the ratio of the scaled intensities collected from the yellow channel to that from the red channel, a sigmoidal plot versus pH is obtained (Fig. 5) which gives a linear relationship between pH 4.2 and 5.6 to enable color coding on a linear scale [18, 24, 25]. A similar plot is obtained for the yellow/green ratio. There is nearly no signal intensity in the yellow channel at pH 6.0, which is consistent with a decrease in fluorescent intensity of the pHAb dye with an increase in pH. The respective green to red channel ratio decreases slightly with pH, consistent with the energy transfer from the green to the yellow channel and the accuracy of the data is influenced by low signal to noise at low probe concentration and differences in imaging conditions (e.g., focus shift), that is only partially accommodated by ratiometric methods, as well as the lack of distinction between particles attached to the outside of the cell, directly exposed to the buffered cell media and the aggregates inside the cell. Some of these limitations have also been encountered in previously reported ratiometric pH sensors [33].

Nevertheless, the data show promise for further development of the experimental method to select and separate the environments for data collection in order to gain spatial information as well as average information as seen here. From the work of Huefner et al. [46] on AuNPs, longer incubation of UCNPs (48 h) with SY5Y cells might increase the cellular uptake (possibly by 400%). Nevertheless, these results indicate that the UCNP-pHAb conjugates give a resolution of ~ 0.5 unit pH (according to the method of Sedlmeier et al. [51])

**Fig. 6:**
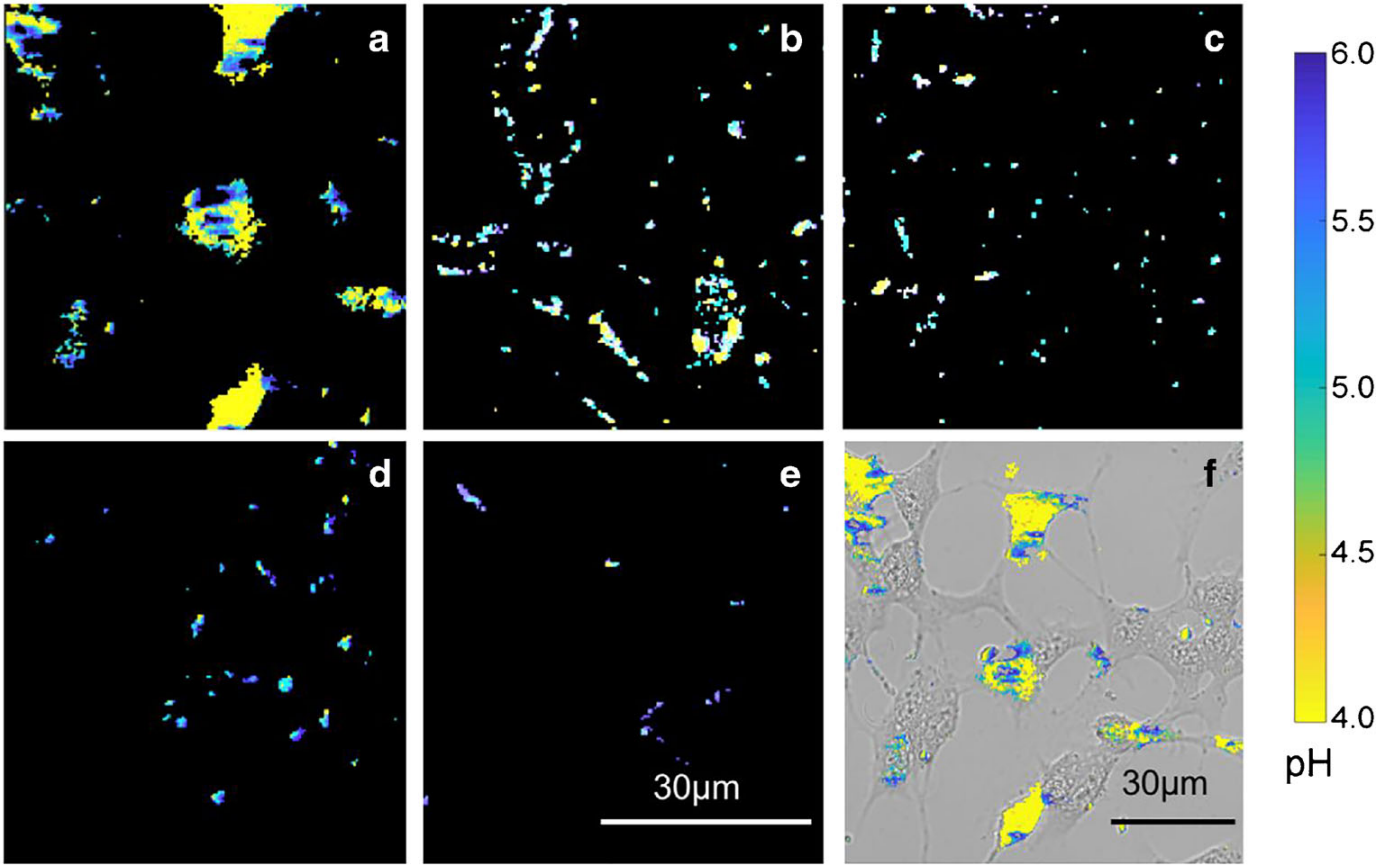
Ratiometric images of UCNP-pHAb in SH-SY5Y cells incubated at pH **a** 4.0, **b** 4.5, **c** 5.0, **d** 5.5, **e** 6.0, and **f** overlay of (**a**) on the transmission image, showing the cell outlines obtained through multiphoton confocal microscopy. The colorbar shows the pseudocolor change with pH. Scale bar = 30 μm. pH estimated from y/r ratio calibration in Fig. 5

Using calibrated color coding, the ratiometric images of UCNP-pHAbs can then be followed, in the SH-SY5Y cells, at the various media pHs (Fig. 6) without using nigericin H^+^/K^+^ ionophore to equilibrate pH across the cell membranes. Media exchange was done to remove excess UCNPs that have not transferred into or on to the cell. The positioning of the particles appeared to be associated with the cells. (compare, for example, Fig. 6 a and f). If the majority of remaining UCNPs imaged are inside the cells, this confirms the impact of pH on the number of particles that manage to get into the cells. pH-dependent uptake of UCNP-pHAbs is expected as a result of the change of surface charge on the UCNP-pHAb, with the number of particles entering the cell decreasing with an increase in pH. However, irrespective of the number of particles, data in Fig. 6 suggest that the UCNP-pHAbs experience a decrease in pH in line with a decrease in buffer pH but with some variance compared with the medium. Indeed, while at pH 6.0, the UCNPs in the cells mainly report pH > 5.6, at the other end of the pH range (pH 4.0), there is a high return of pH 4.0 but also intermediate values as high as 5.5. At intermediate pHs, there is a distribution of pHs reported in the cells from 4.4 ≤ pH ≥ 5.6. Nevertheless, the trend shows an increase in low pH reporting particles with a decrease in pH of the cell environment.

### Localization of the UCNP-pHAb in SH-SY5Y cells

The indication of a lowering of pH in the environment of the UCNP in the cell is also anticipated in endocytosis. This describes the internalization of extracellular material, as seen here, whereby a portion of plasma membrane surrounds the material, then a vesicle with the ingested content buds off inside the cell [52]. In general, particles that are <5 μm in size can be internalized by cells through endocytosis [53]. Because UCNP-pHAb is of a suitable size distribution and has an excess of free amines to give the particles a net positive charge, most adherent cell lines are expected to spontaneously take them up by endocytosis. The cellular fate of the UCNP-pHAb probe was explored by staining with LysoBrite dye. Generally, studies on colocalization of a pH probe and lysosome have sought qualitative spatial experiment data [25, 54, 55], observing whether there was evidence that reflected the combined contributions of both probes when the microscopy images were superimposed [25, 38]. However, colocalization has two components: co-occurrence (spatial overlap of two probes) and correlation (proportional codistribution within and between structures) [38]. In this case, the UCNP-pHAb would be expected to overlap with LysoBrite if the probe localized in lysosomes (co-occurrence), but the UCNP-pHAb would not be expected to enter every lysosome in the cell (correlation). It would not be unusual to see some lysosomes with UCNP-pHAb and others without, so the colocalization component of interest here is co-occurence.

**Fig. 7:**
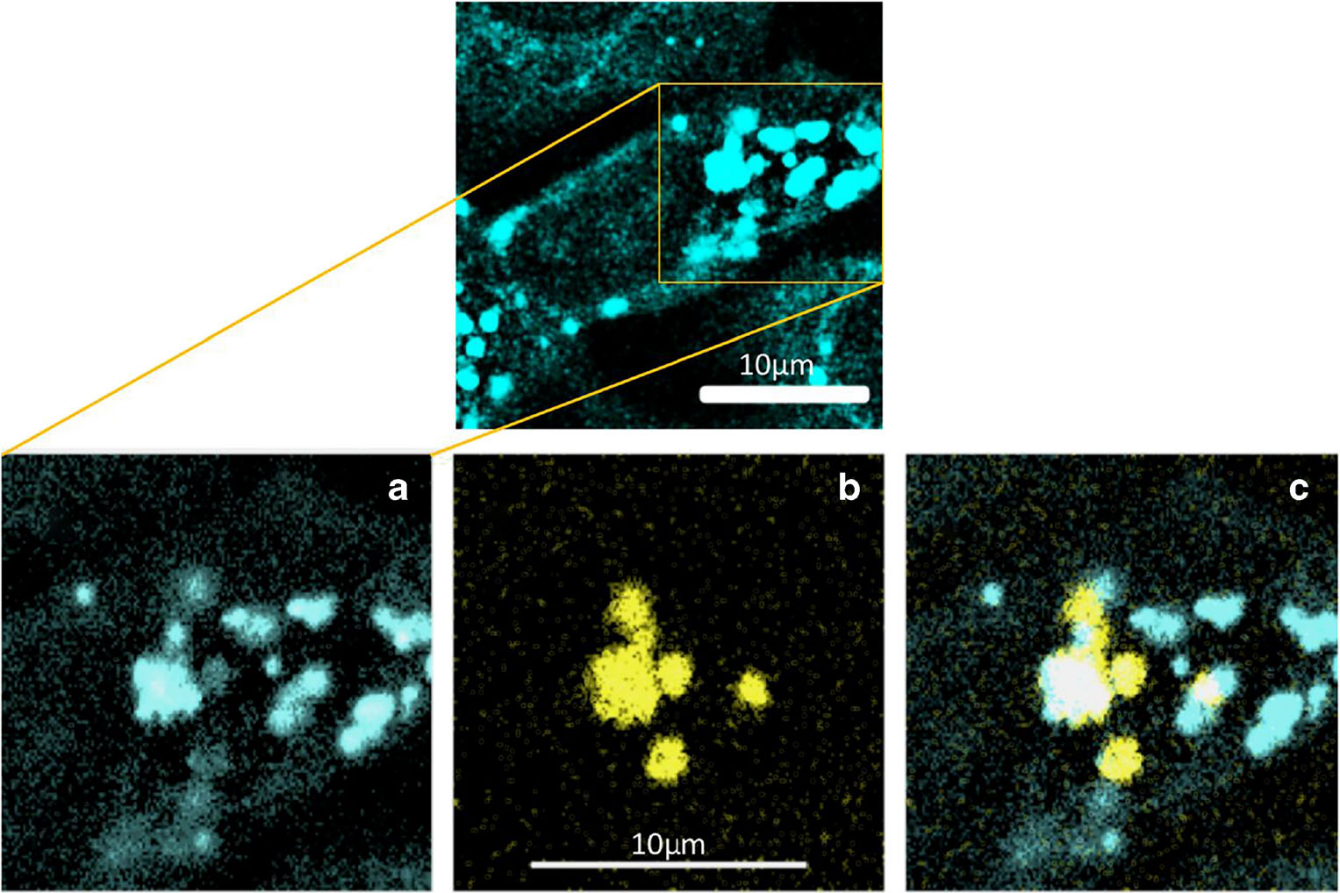
Example images of SH-SY5Y cells incubated with UCNP-pHAb and LysoBrite. Images (**a**)–(**c**) are magnified from the header image showing the LysoBrite channel and locating the particles in a cell **a** shows the LysoBrite channel under 405 nm excitation and **b** shows the pHAb channel under 532 nm excitation. **c** is the merged image of (**a**) and (**b**)

Visual evaluation of colocalization suggests that the UCNP-pHAb and LysoBrite associate with the same structure in some cases (Fig. 7). It also appears that some of the lysosomes in the cell do not contain UCNP-pHAb. Quantitative colocalization analysis can calculate the relative distribution of the two probes and determine whether the degree of colocalization exceeds random chance [38, 39].

Manders’ colocalization coefficient (MCC) measures co-occurrence independent of correlation, so it is an appropriate measure when one probe localizes in more compartments relative to another probe. When used here, MCC calculates the fraction of pHAb with LysoBrite and the fraction of LysoBrite with pHAb. MCC is very sensitive to the background because the calculation does not subtract out the channel mean intensity, so the background values need to be identified and subtracted from the images before any calculations are performed. The Costes thresholding method is widely used to eliminate background, but it fails when there are significant differences in the number of compartments labeled with each fluorophore. Median subtraction does not encounter this problem and has been shown to work for dispersed objects like endosomes [38], so it is applied here.

To obtain the median-subtracted intensity (ESM Fig. S2), the local median intensity was subtracted from the original intensity at each pixel along a line drawn through one cell (> 10,000-pixel data points were recorded on each sample) The median subtraction is quite effective at removing the background noise in Fig.7. The original pixel intensities (indicated by the green lines in Fig. S2) for a typical line through a cell do not reach a gray value of zero, even in places where the images appear black in Fig. 7. The local median intensity of a square pixel region captures the low background variation (black lines). Subtracting the median intensities from the original intensities gives intensity profiles (red lines) that match the trend of the original image profiles while successfully isolating the signal from the background. These median-subtracted intensities were then used to calculate MCC values that quantify the fractional overlap between the LysoBrite and pHAb dyes.

The resulting MCC values after median subtraction are shown in Table S1 (see ESM). The *MCC*_*1*_ values suggest that not all lysosomes contain UCNP-pHAb, while the *MCC*_*2*_ values indicate that the vast majority of the UCNP-pHAb probes are in lysosomes. Significance testing was performed to determine whether UCNP-pHAb colocalizes with LysoBrite dye more than what would be expected by chance. Because every cell has a different proportion of cyan pixels to begin with, each *MCC*_*expected*_ will also be different under the null hypothesis of no colocalization. The *t* test needs to be done on the difference (*MCC*_*diff*_) between *MCC*_*observed*_ and *MCC*_*expected*_ (Table 2). Student’s one-sample, one-tailed test to compare the mean *MCC*_*diff*_ to 0 gives *p* < 0.0001 (t = 14.5, d.f. = 4, *α* = 0.05), confirming that the UCNP-pHAb localizes in lysosomes. The small fraction of UCNP-pHAb not in these compartments (complement of fractions in the second column, Table 2) may be in early endosomes or late endosomes instead, which matches the pHs shown in the ratiometric image (turquoise and violet areas in Fig. 6).

**Table 2:** MCC values for one-tailed, one-sample *t* test to determine colocalization

*MCC*_*expected*_	*MCC*_*observed*_	*MCC*_*diff*_
0.118	0.957	0.839
0.172	0.868	0.696
0.071	0.748	0.677
0.105	0.917	0.812
0.113	0.679	0.566

### Endocytosis pathway studies

Phagocytosis and pinocytosis are the two categories of endocytic pathways that enable cells to internalize particles. Phagocytosis is largely limited to immunogenic cells, but the latter includes macropinocytosis, caveolae-mediated endocytosis, and clathrin-mediated endocytosis, which differ with respect to the protein coat, size, and fate of the vesicles [55]. Although it is still a challenge to predict the uptake mechanism based on a given nanoparticle property, size is considered to be the major factor [55, 56]. Macropinocytosis involves particles >1 μm, caveolae-mediated endocytosis is limited to particles < 100 nm, and materials < 200 nm can participate in clathrin-mediated endocytosis to enter the endosomal/lysosomal trafficking route [55, 57]. Given the distribution of particle sizes from DLS, clathrin-mediated endocytosis appears to be the most likely uptake mechanism for the nanosensor.

Drug inhibitor studies were performed with flow cytometry to confirm the mechanism by which the UCNP-pHAb nanosensor is taken up by the SH-SY5Y cells. To first confirm that the flow cytometer was able to detect the fluorescent response of pHAb with a PE filter, the dye was directly excited with a 488 nm laser in citrate/phosphate buffer at two different pHs. At pH 4, the flow cytometer detected higher fluorescence from the UCNP-pHAb compared with the particles at pH 6 (ESM Fig. S3). This matched the expected pH-dependent emissive response of pHAb from Fig. 1.

Chlorpromazine (CPZ) and nystatin (NYS) were selected as drug inhibitors because they interfere with clathrin-mediated endocytosis and caveolae-mediated endocytosis, respectively, and have been used previously in endocytosis studies with SH-SY5Y cells. CPZ stops AP2 adapter protein from binding to clathrin-coated vesicles [58], and NYS binds to the cholesterol that is needed to maintain the structural integrity of the caveolae coat [59]. If the drug induces a significant decrease in intracellular pHAb fluorescence, that is an indication that the corresponding endocytic mechanism plays a significant role in the uptake of the nanosensor.

**Fig. 8:**
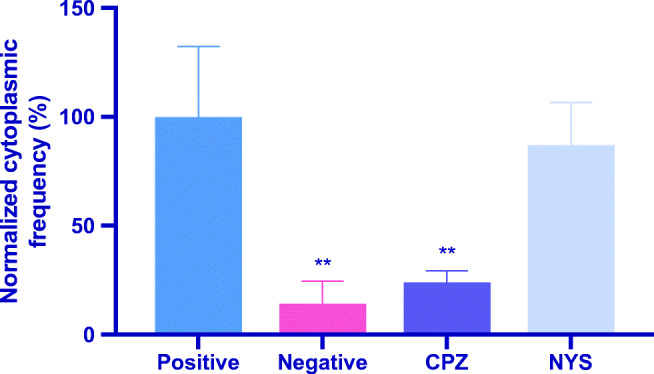
Effects of pharmacological inhibitors on UCNP-pHAb uptake after 1.5 h of incubation. The samples were measured with flow cytometry, and each experiment was repeated three times. Statistical significance was calculated with one-way analysis of variance (ANOVA) and Dunnett’s multiple comparison test compared with the positive control. **P ≤ 0.01

The negative control (media only), CPZ, and NYS groups were normalized to the positive control of UCNP-pHAb (Fig. 8). The intrinsic fluorescence of the SH-SY5Y cells was negligibly low; any higher measurement of fluorescence could be attributed to the internalized pHAb. Incubation with CPZ showed a ~ 75% decrease in fluorescence, indicating the importance of clathrin-mediated endocytosis in the uptake of these nanosensors. Incubation with NYS showed ~ 10% decrease in fluorescence, which was not statistically significant and suggested that caveolae-mediated endocytosis was not an essential method of uptake. This information provides initial data to develop the UCNP-pHAb nanosensor to be used to track the pH for drugs during uptake via clathrin-mediated endocytosis [60].

## Conclusions

This work examined the design and application of a ratiometric intracellular pH imaging probe based on UCNPs and pHAb dye. The dual-wavelength nanosensor was based on the ratio of the sensitized yellow emission from pHAb and the reference red emission from UCNP. Intracellular calibration of the probe was performed after treatment with nigericin, which equilibrated the internal pH to that of the extracellular environment. After this calibration, the pH sensors were used to measure the pH of compartments inside the cell and suggested quite a heterogeneous pH profile.

The UCNP-pHAb sensor was non-toxic to SH-SY5Y cells for the duration of the experiments. Preliminary colocalization and endocytosis pathway studies suggested that the nanosensor was taken up mostly by clathrin-mediated endocytosis and localized in lysosomes, making them ideal probes to study vesicle acidification for its effect on drug degradation following uptake. These probes work well for detecting pH changes in in vitro cultures due to their strong pH-dependent fluorescence and may also prove useful in more complex 3D models such as organoids. The results were also promising for more advanced optical imaging techniques and particle tracking studies, suggesting that upconversion-based pH nanosensors can be used to study live cells when they are exposed to different stimuli that induce pH change.

## Electronic supplementary material

ESM 1 (PDF 280 kb)
