# Supplementary material for: Upconversion nanoparticles as intracellular pH messengers
Source: Anal Bioanal Chem. 2020 Jul 2;412(24):6567–81. doi: 10.1007/s00216-020-02768-5 (PMC7442772; doi:10.1007/s00216-020-02768-5)
Supplement: Supplementary file 1 — (PDF 280 kb) [file 216_2020_2768_MOESM1_ESM.pdf]

Analytical and Bioanalytical Chemistry

Electronic Supplementary Material

Upconversion nanoparticles as intracellular pH messengers

Evaline S. Tsai, Fadwa Joud, Lisa M. Wiesholler, Thomas Hirsch,
Elizabeth A. H. Hall

Synthesis and Surface Modification of UCNPs

For the synthesis of 1 mmol of β-$NaYF_4$: 20% Yb, 2% Er particles, the corresponding molar ratios of lanthanide chloride hexhaydrates were dissolved in methanol (5 mL) then mixed with oleic acid (8 mL) and 1-octadecene (15 mL). The resulting suspension was heated to 160 °C, put under vacuum for 30 min to form a clear solution, then subsequently cooled to room temperature. 4.0 mmol of ammonium fluoride and 2.5 mmol of sodium hydroxide were dissolved in methanol and added to the original solution. Afterwards, the mixture was kept at 120 °C for half an hour then heated to 325 °C. The progress of the reaction was monitored with a 980 nm cw laser module (200 mW). Eight minutes after the green upconversion luminescence could be observed by the naked eye, the suspension was cooled to room temperature. For purification, the pellet was washed twice with chloroform/ethanol (1:10 v/v) and three times with cyclohexane/ethanol (1:10 v/v). The particles were redispersed in cyclohexane (10 mL) and centrifuged (1000 g, 3 min) to remove aggregates and produce the final suspension.

The above steps were followed to make α-$NaYF_4$ particles as well, except during the last heating step, the temperature was set to 240 °C for 30 min. The β-$NaYF_4$: Yb, Er particles (core material) and α-$NaYF_4$ particles (shell precursor) were dispersed in cyclohexane inside two separate flasks. 5 mL of oleic acid and 5 mL of 1-octadecene were added to each. Both flasks were heated to 100 °C then placed under vacuum for 30 min to vaporize the cyclohexane. The hexagonal phase particles were brought to 325 °C, while the cubic phase particles were maintained at 100 °C. Every 10 min, <3 mL of the shell precursor solution was injected into the other flask. The volume was kept low to prevent the temperature from falling below 300 °C. Once the precursor solution was completely transferred, the resulting suspension was allowed to cool to room temperature. The redispersion-precipitation-centrifugation steps described above were used to purify and precipitate the core-shell UCNPs. This procedure can be scaled to create batches up to 20 mmol of core-shell UCNPs.

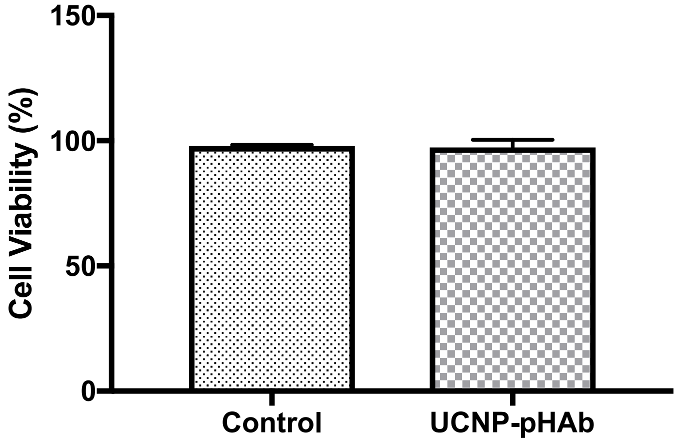

Fig. S1 Cell viability with trypan blue exclusion assay. The control had 98 ± 1% cell viability and the cells incubated with UCNP-pHAb (0.01 mg/mL) had 97 ± 3% cell viability. The SH-SY5Y cells did not show significant decrease in cell viability after 24 h of incubation with 0.01 mg/mL UCNP-pHAb

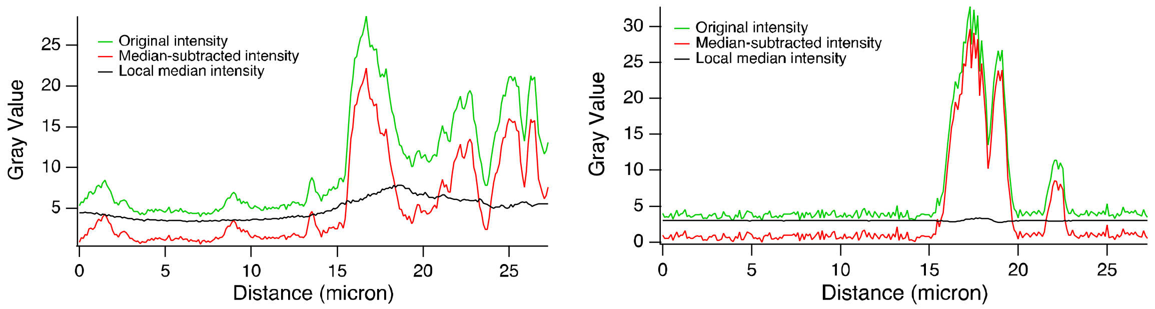

Fig. S2 Pixel intensities along a line drawn through the cell in Figure 4.19. The LysoBrite channel is on the left and the pHAb channel is on the right

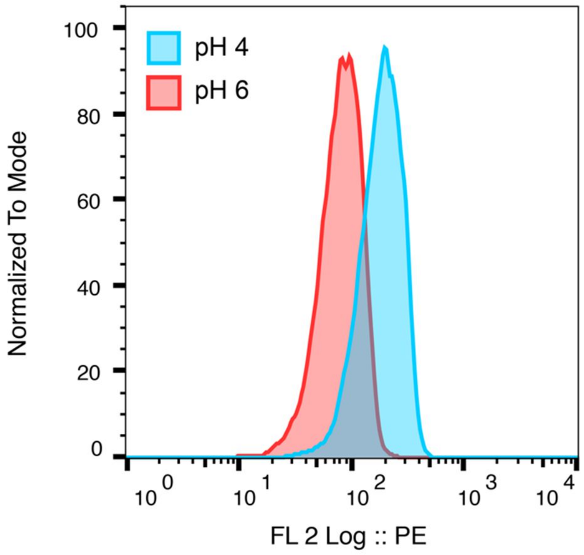

Fig. S3 Fluorescence histogram of UCNP-pHAb at pH 4 and 6 from flow cytometry. Excitation was at 488 nm to directly excite the dye, and a PE filter was chosen because it overlapped well with the emission of the dye

Table S1 MCC values for pHAb and LysoBrite in cells after median subtraction

MCC_1 (fraction of cyan with yellow)	MCC_2 (fraction of yellow with cyan)
0.18	0.96
0.39	0.87
0.10	0.75
0.31	0.92
0.19	0.68
